# Accumulation of myeloid lineage cells is mapping out liver fibrosis post injury: a targetable lesion using Ketanserin

**DOI:** 10.1038/s12276-018-0118-x

**Published:** 2018-07-19

**Authors:** Saeid Amini-Nik, Ali-Reza Sadri, Li Diao, Cassandra Belo, Marc G. Jeschke

**Affiliations:** 1 Sunnybrook Research Institute, Toronto, Canada; 2 Laboratory Medicine and Pathobiology, University of Toronto, Toronto, Canada; 3 Department of Surgery, Division of Plastic Surgery, University of Toronto, Toronto, Canada; 4 Institute of Medical Science, University of Toronto, Toronto, Canada; 5 Department of Immunology, University of Toronto, Toronto, Canada; 6 Ross-Tilley Burn Centre, Sunnybrook Health Sciences Centre, Toronto, Canada

**Keywords:** Mechanisms of disease, Acute inflammation

## Abstract

Liver fibrosis is problematic after persistent injury. However, little is known about its response to an acute insult. Accumulation of myeloid lineage cells contributes into the promotion and resolution of inflammation and fibrosis. Using Cre-transgenic mice that specifically mark myeloid lineage cells with EYFP and burn as a model of acute systemic injury, we investigated the role of myeloid lineage cells in the liver after acute injury. Our data show that thermal injury in mice (30% total body surface area) induces fibrosis predominantly around portal venules whereas myeloid cells are enriched throughout the liver. The fibrosis peaks around 1–2 weeks post injury and resolves by week 3. Ablating myeloid cells led to lower fibrosis. Through FACS sorting, we isolated myeloid lineage cells (EYFP +ve cells) from injured animals and from the control uninjured animals and subjected the extracted RNA from these cells to microarray analysis. Microarray analysis revealed an inflammatory signature for EYFP +ve cells isolated from injured animals in comparison with control cells. Moreover, it showed modulation of components of the serotonin (5-HT) pathway in myeloid cells. Antagonizing the 5HT_2A/2C_ receptor decreased fibrosis in thermally injured mice by skewing macrophages away from their pro-fibrotic phenotype. Macrophages conditioned with Ketanserin showed a lower pro-fibrotic phenotype in a co-culture system with mesenchymal cells. There is a spatiotemporal pattern in liver fibrosis post-thermal injury, which is associated with the influx of myeloid cells. Treating mice with a 5HT_2A/2C_ receptor antagonist promotes an anti-fibrotic effect, through modulating the phenotype of macrophages.

## Liver damage: Injury-induced fibrosis

A drug that affects serotonin pathway in the liver following systemic injury could help limit damage caused by fibrosis. Severe burn injuries can result in liver fibrosis, the over-production of connective tissues promoted by pro-inflammatory immune cells, including those derived from bone marrow myeloid cells. Chronic fibrosis can cause tissue dysfunction and extensive scarring which can contribute into liver dysfunction. In experiments on mice, Saeid Amini-Nik and Marc Jeschke at the University of Toronto, Canada, and co-workers demonstrated that myeloid cells are enriched in the liver after thermal injury, with a phenotype reminiscent of inflammatory macrophages. Treating the mice with a drug called Ketanserin, which targets serotonin pathway, altered the myeloid-derived immune cells so that they were less likely to cause fibrosis. This suggests a possible therapy for liver fibrosis post-injury.

## Introduction

Severe burn injury results in a systemic response with substantial hepatic alterations^1^. The liver has a diverse role in response to a thermal injury, such as producing acute phase proteins and regulating the systemic inflammatory response^1^. Although several reports show there is a hypermetabolic response, increased inflammation, and endoplasmic reticulum (ER) stress in the liver post-thermal injury^2,3^, the spatial pattern and nature of this damage are still a mystery.

Upon liver injury, there is a wound-healing response that involves extracellular matrix (ECM) deposition^4^. This is a vital reaction for the liver to protect or repair itself ^5^. It has been shown that after an acute injury there is fibrosis, which works to shield hepatocytes from toxins rather than having any detrimental role^6^. However, clinically fibrosis is an undesirable event and seen as a form of pathology.

For a long time macrophages have been known to be the promoters of fibrosis but more recently have been shown to be essential for fibrosis resolution as well^7–9^. Macrophages have numerous functional states, which in vitro are normally labeled as M1 (classically activated) macrophages or M2 (alternatively activated) macrophages. M1 macrophages have pro-inflammatory functions whereas M2 macrophages promote resolution of inflammation and wound healing^10,11^. Though, the classification of macrophages in vivo is not as black and white. Macrophage heterogeneity is highly complex, as these cells can switch between phenotypes depending on the environmental cues, making it difficult to fully characterize these cells in vivo. Thus, it is best to classify these cells based on functionality rather than marker-based phenotype. Due to their programming versatility and essential role in all stages of wound healing, macrophages have become a therapeutic target for inflammatory and fibrotic conditions^12,13^.

One potential therapeutic remedy for liver injury that is currently being investigated in the field is serotonin (5-hydroxytryptamine, 5HT). Primarily known as a neurotransmitter, 5HT, has an essential role in mood, cognition, feeding, and sleep^14^. However, 90% of 5-HT is produced outside the CNS, mainly found in the gut^15^. The role of 5-HT includes cell proliferation, vascular contraction and relaxation, apoptosis, and platelet aggregation. Current studies on rodents and humans suggest that 5-HT has an imperative role in liver regeneration and fibrosis^16–19^.

Here, using thermal injury as a model of systemic injury and Cre-transgenic myeloid reporter mice, we show that there is a spatiotemporal pattern in liver fibrosis post-thermal injury and myeloid lineage cells orchestrate this fibrotic response. Treating mice with Ketanserin decreases portal fibrosis possibly by skewing the phenotype of myeloid cells away from their pro-fibrotic form. To the best of our knowledge, this is the first study to look at fibrosis in the liver post-thermal injury and show a spatiotemporal pattern of fibrosis after severe trauma.

## Materials and methods

### Mice

We used our previously reported myeloid lineage reporter mice^7^. Briefly, generation of myeloid lineage reporter mice was done using the Cre-loxP system. To drive Cre in Lysz positive cells only, we used the Lysz-Cre (B6.129-Lysztm1(cre)Ifo/J) mice. Our reporter gene is derived from ROSA-EYFP (B6.129 × 1-Gt[ROSA]26Sortm1(EYFP)Cos/J) mice, which contain an EYFP gene inserted downstream of a floxed stop codon. Breeding these mice with mice expressing recombinase results in excision of the floxed stop codon and expression of EYFP. The progeny of these mice are called Lysz-Cre;ROSA-EYFP in this manuscript.

### Experimental protocol (burn)

Animal procedures were reviewed and approved by Sunnybrook Research Institute and Sunnybrook Health Sciences Centre at University of Toronto animal care and use committee. Mice were subjected to a full-thickness scald burn. Briefly, animals were anesthetized with inhaled isofluorane and received an intraperitoneal (IP) injection of the analgesic buprenorphine. The dorsum of the animal was shaved and lactated ringer’s solution was IP injected along the spine. A 30% total body surface area (TBSA) thermal injury was induced by placing the animal on a mold that exposes the dorsum^20^. The dorsum of the animal is then exposed to water pre-heated to 98 °C. The exposure lasts for 10 s. The animals are placed in separate cages post-thermal injury. Sham animals received the prep but did not receive a thermal injury.

### Histological examination

Masson’s Trichrome and immunohistochemistry was done as described before^21,22^. For immunofluorescence staining, liver sections were blocked (1% bovine serum albumin in 0.5% PBS-TritonX) and incubated in primary antibodies: F4/80 (AbD Serotec), GFP (Rockland), and BrdU (Cell Signaling). The primary antibodies were detected using AF488 (Vector) and AF647 (Vector). Proliferation in hepatic cells was measured using BrdU uptake. Mice received an IP injection of BrdU 24 h prior to death (50 mg/kg of animal weight).

### Image acquisition and image analysis

Image capture of liver sections stained via immunofluorescence was done using the Zeiss observer Z1 spinning disc confocal microscope. Sections stained via immunohistochemistry were imaged using the Zeiss Axiovert 200 light microscope at 20× and 40× magnification for quantification.

### Fibrosis index

Photoshop CS5 was used to measure collagen in the portal zone. We used the color range option to measure blue pixels in the uploaded tiff file image of liver sections stained using Masson’s Trichrome protocol. We then did a pixel count of the area of the portal venule, which was used to get a ratio (pixel count of collagen/pixel count of portal venule area) to control for vessel size. The ratio was multiplied by 1000 giving us the fibrosis index.

### Western blotting

Antibody against Desmin (PA5-16705) was purchased from Thermo Fisher Scientific (Rockford, IL, USA); F4/80 (ab186073) was purchased from Abcam (Cambridge, MA, USA); and GAPDH (14C10, #2118) was purchased from Cell Signaling (Danvers, MA, USA). Clarity Western ECL substrate was purchased from Bio-Rad (Hercules, CA, USA). Liver homogenates (50 μg of protein per well) were separated by 10% SDS-PAGE gel, proteins were transferred to nitrocellulose membrane, and then blots were probed using the antibodies listed above. Band intensities were detected, normalized and quantified with the Chemidoc and Image Lab 5.0 software (Bio-Rad Laboratories, Hercules, CA). GAPDH was used as loading control.

### Clodronate

Liposomal clodronate was purchased from Dr. Nico van Rooijen (www.clodronateliposomes.org). They were prepared as previously described van^23^. Wild-type C57/BL6 mice were purchased from Jackson laboratories. These mice received an IP injection of liposomal clodronate (0.1 mL/10 g of animal tissue) or PBS 48 h prior to a thermal injury and 48 h post-thermal injury.

### Flow cytometry and microarray analysis

Mice with the same genotype (Lysz-Cre;ROSA-EYFP) were exposed to a 30% thermal injury or had sham preparation. They were monitored for 1 week prior to harvesting. The liver was removed from each mouse and digested with collagenase cocktail (1000 U/ml collagenase, 1X dispase, 0.05% trypsin-EDTA in DMEM with 1% Ab/Am) for 30 min at 37 °C. For isolation of EYFP+ cells, half of the dissociated cells were taken from each mouse, mixed and subjected to BD FACS ARIA II for flow cytometry analysis and sorting of the cells. The EYFP+ population was sorted and sham provided 600,000 cells and burn provided 1,300,000 cells. These cells were stored at −80 °C until RNA preparation. The remaining half of the liver were sampled through flow cytometry separately and probing for EYFP, CD11b (eBioscience, APC-eFluor 780) and F4/80 (Biolegend, PerCP cy5.5). The flow cytometer used for cell analysis was a Cytek DxP FACSCalibur. For RNA Preparation TRIzol reagent (Life Technologies) was used to lyse the cells. The manufacturer’s instructions were followed. In short, 1 ml of TRIzol was added to each cell pellet. Chloroform was added and the samples were centrifuged at 12,000*g* for 15 min at 4 °C. The aqueous phase was carefully removed and transferred to a new tube. RNA was precipitated with 100% isopropanol and centrifuged at 12,000*g* for 10 min at 4 °C. The supernatant was removed and the RNA pellet was washed with 75% ethanol, centrifuged at 7500*g* for 5 min at 4 °C. The pellet was air dried and resuspended in RNase-free water. The absorbance at 260 and 280 nm was measured to determine the RNA yield. For cDNA synthesis, 2 μg of RNA was taken and used with the kit High-Capacity cDNA Reverse Transcription Kit (Life Technologies) according to the manufacturer’s instructions.

### Ketanserin treatment

Wild-type C57BL/6 mice were treated with 10 mg/kg of Ketanserin daily for 7 days prior to the thermal injury and treated for another 14 days post-thermal injury. For in vitro experiments, Ketanserin was diluted in sterile saline. Bone-marrow-derived macrophages (BMDMs) were cultured for 7 days. On day 6 and 7 macrophages were treated with 10^–9^ M of Ketanserin.

### Cell culture

BMDMs were retrieved from wild-type C57BL/6 mice. Cells were cultured in macrophage medium consisting of RPMI-1640, 10% fetal bovine serum, 10% L929, and 1% antibiotics-antimicrobial solution. Cells were cultured at 37 °C in a humidified atmosphere with 5% carbon dioxide for 7 days.

### Co-culture of macrophages and BMD-MSCs

FalconTM cell culture inserts for 6 well plates were used to co-culture BMDMs with BMDMSCs. Macrophages and MSCs were first cultured in separate 6-well plates for 7 days to allow maturation of cells. Macrophages were cultured in macrophage media, which is described above. MSCs were cultured in MSC conditioned media consisting of MesenCult™ MSC Basal Medium (Mouse), Mesenchymal Stem Cell Stimulatory Supplements (Mouse) and MesenPure™. Macrophages are added to the wells and inserts. On days 6 and 7 macrophages are treated with Ketanserin or saline and on day 10 inserts with macrophages pretreated with Ketanserin or saline are added to six well plates with MSCs at the bottom. The two cell types are co-cultured for 3 days.

### Phagocytosis assay

Fluorescently labeled zymosan particles (Life technologies) were centrifuged onto the BMDMs at 450 g for 2 min. The samples were then incubated at 37 °C, 5% CO_2_ for 1 h. Phagocytosis was stopped by ice-cold PBS followed by four washes and cells fixed in 4% PFA for 20 min. This was followed by two more washes with PBS. Cells were permeabilized with PBS containing 1% BSA and 0.5% Triton X-100 for 15 min. Cells were washed three more times with PBS containing 1% BSA. Cells were then stained with FITC-labeled phalloidin and Dapi.

### RNA isolation and real-time quantitative polymerase chain reaction

TRIzol reagent (Invitrogen, Carlsbad, CA, USA) was used to isolate RNA according to manufacturer’s instructions. The NanoDrop-2000 spectrophotometer (ThermoScientific, Waltham, MA, USA) was used to determine total RNA yield. cDNA synthesis was done in a thermocycler (AB Applied Biosystems, Foster City, CA, USA), after mixing 1 μg RNA and a master mix prepared with the high-capacity cDNA synthesis reverse transcription kit (AB Applied Biosystems). SYBR® Green PCR Master Mix (Applied Biosystems) was used to perform a Quantitative polymerase chain reaction (PCR) looking at the following genes of interest: *Il-6, Il-10, Tgf-β1, Tgf-β3, Lcn-2, iNos, Arg*, and *Tnf-α*.

### Analysis of liver function

Prior to sacrificing of mice, 0.5 mL of blood was collected and serum was separated by centrifugation at 3000 rpm for 20 min. Serum AST and ALT levels were measured using AST and ALT Activity Assay Kits (Sigma-Aldrich).

### Statistical analysis

Statistical comparisons between the groups were performed using an unpaired student’s *t*-test. A two-tailed *p*-value ≤ 0.05 was considered significant. Data were graphically expressed as the mean of the target group ± the standard error of the mean.

## Results

### Thermal injury results in liver fibrosis with a unique spatiotemporal pattern

**Fig. 1:**
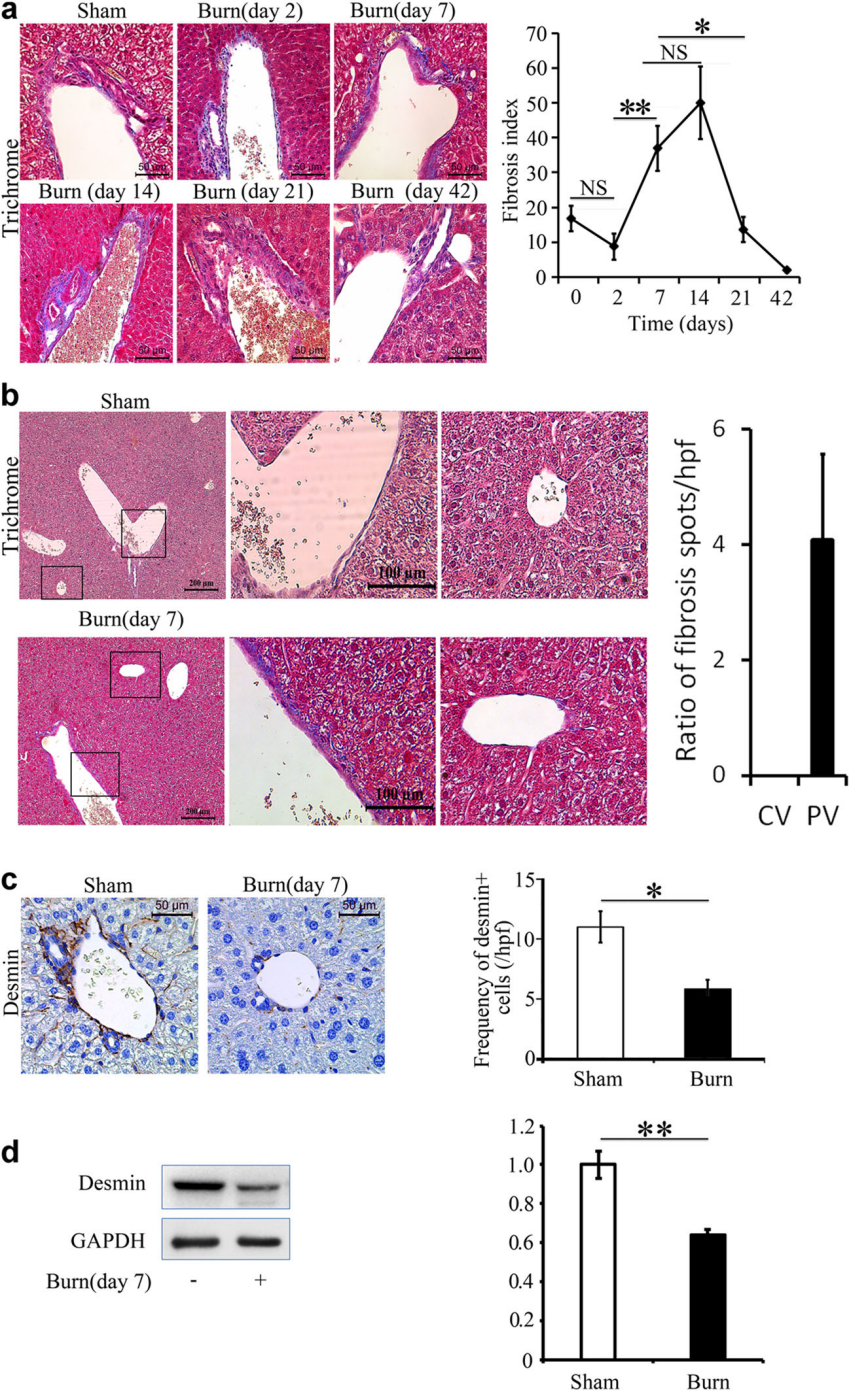
**Thermal injury primes liver fibrosis in a spatiotemporal pattern**. **a** There is a temporal trend in fibrosis around PVs post-thermal injury. Mice were examined after 2, 7, 14, 21, and 42 days post-thermal injury; *n* = 5–7, scale bar 50 μm, *P < 0.05, **P < 0.001. NS not significant. **b** Trichrome staining shows that collagen is deposited predominately around PVs rather than CVs. **c-d** Thermal injury promotes a reduction in desmin+ cells *n* = 3–5

Knowing that there is a systemic inflammatory response and profound liver dysfunction post-thermal injury; we investigated whether thermal injury induces a fibrotic response in the liver. A mild temporal pattern in liver fibrosis was observed (Fig. 1a). After a 30% TBSA full-thickness burn, mice were sacrificed at 2, 7, 14, 21, and 42 days post-thermal injury. The comparison was made between age-matched uninjured litter-mates (control sham group). Liver fibrosis was assessed using Masson’s Trichrome staining. Hepatic fibrosis peaked between 7 and 14 days post-thermal injury, while its resolution occurred by day 21 and remained low at day 42 (Fig. 1a). The observed fibrosis was comparable to the F1 stage of the metavir score, portal fibrosis without septae^24^. Interestingly, the observed fibrosis is found only in the portal field, predominantly around portal venules (PV) rather than around the central veins (CV) (Fig. 1b). In addition, we observed a decrease in quiescent hepatic stellate cells (desmin+ cells), the resident mesenchymal stem cells of the liver, after thermal injury (Fig. 1c, d). This suggests that they have transitioned into an activated state as myofibroblasts, the key producers of collagen.

### Myeloid lineage cells accumulate in the liver post-thermal injury

**Fig. 2:**
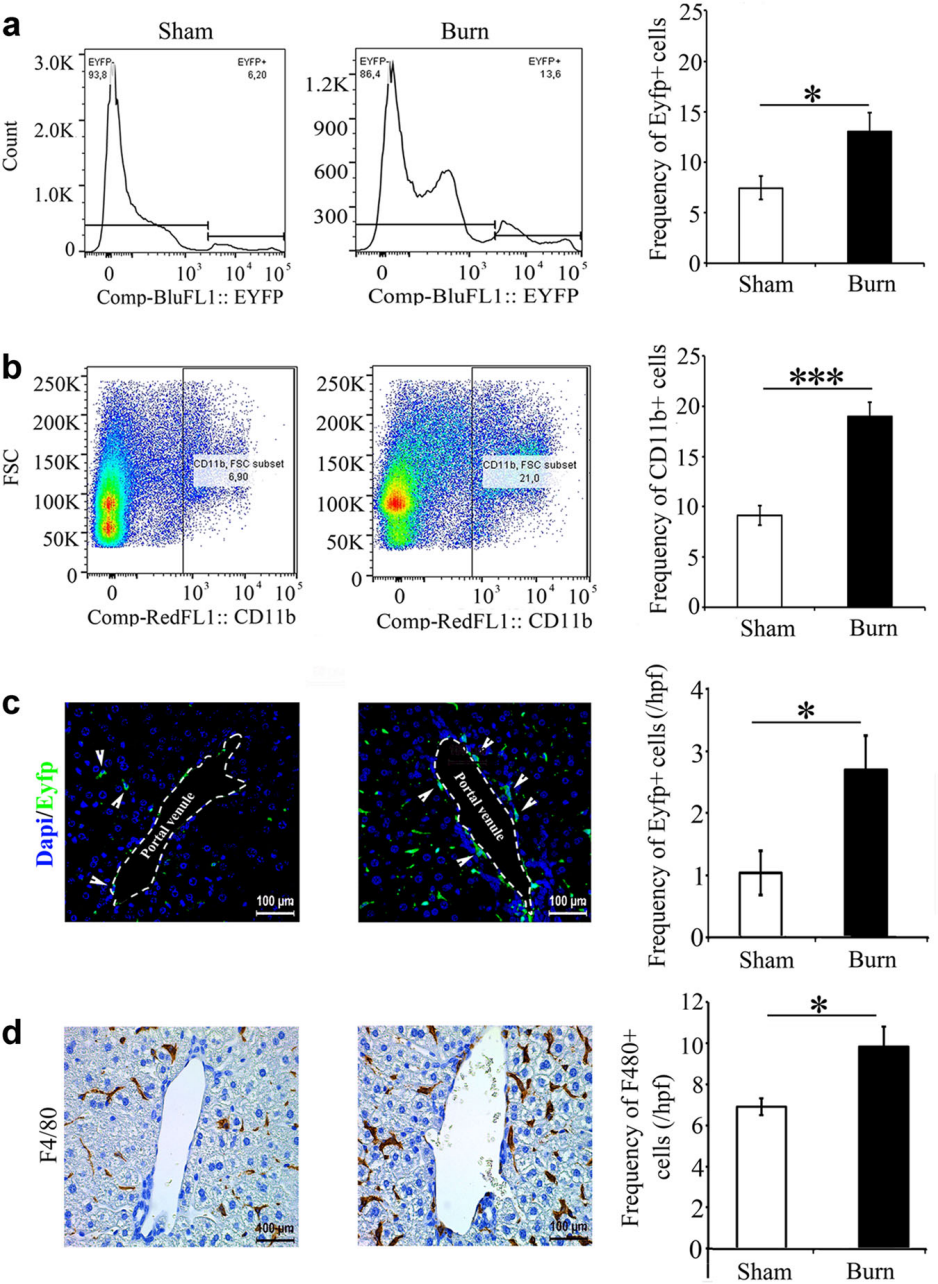
**Myeloid cells are recruited to the liver post-thermal injury**. **a**, **b** Flow cytometry shows an increase in EYFP+ and cd11b+ cells. **c** Immunofluorescence of Lysz-Cre ROSA-EYFP livers shows an enrichment of EYFP+ cells post-thermal injury, in particular around PVs. scale bar 100 μm. **d** Immunohistochemistry staining for F4/80+ cells show an increase post-thermal injury in these cells in the liver and surrounding PV sites; *n* = 5 for all the panels, scale bar 100 μm. *P < 0.05, and ***P < 0.001

Having identified the time when there is maximal fibrosis, at roughly 7 days post-thermal injury, we investigated whether there is an accumulation of myeloid cells in the liver at that time point. Through flow cytometry, we observed a significant increase in EYFP+ cells and cd11b+ cells (Fig. 2a). The percentage of EYFP+ cells that are cd11b+ increases post-thermal injury suggesting that these cells are from an extra-hepatic source (Supporting Fig.1A). Double staining of EYFP and F4/80 in the liver shows that 90% of EYFP+ cells are also F4/80+ (Supporting Fig.1B, C). We also observed an increase in the percentage of EYFP+ cells in the bone marrow post-thermal injury, suggesting that systemic thermal injury mobilizes bone marrow cells and shift bone marrow fate more toward a myeloid lineage (Supporting Fig. 2A and 2B). Immunohistochemical staining showed a significant increase in myeloid cells (Fig. 2b) and F4/80 positive macrophages at 7 days’ post-thermal injury (Fig. 2c). Interestingly, accumulation of EYFP+ cells and F4/80+ cells were observed in the portal field. To find out if the increase in myeloid lineage cells in the liver is due to recruitment or proliferation of EYFP+ cells, we did double staining for EYFP and BrdU. We observed a very limited number of EYFP+ cells that were BrdU+, comparable with sham mice, at 1 and 2 weeks post-thermal injury, suggesting that accumulation of myeloid cells is mainly due to recruitment (Supporting Fig. 1D).

### Ablating macrophages prevents fibrosis in the liver post-thermal injury

**Fig. 3:**
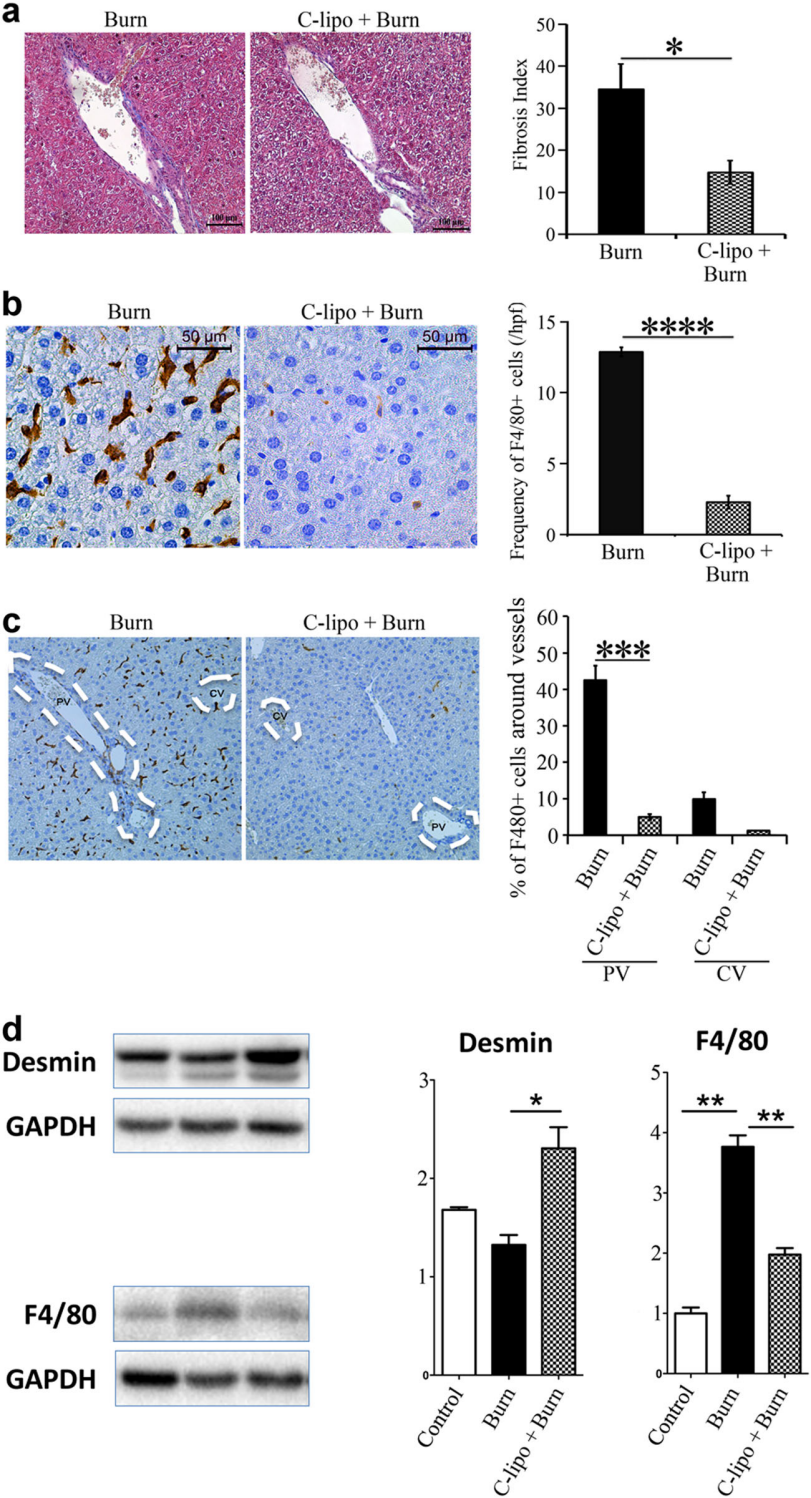
**Myeloid cells have an essential role in the induction of fibrosis post-thermal injury**. **a** Mice treated with liposomal clodronate compared with control treated group. Trichrome staining shows less fibrosis around portal venules post-thermal injury when myeloid cells are depleted; *n* = 9, scale bar 50 μm. **b** Immunohistochemistry shows a highly effective ablation of F4/80+ cells in liposomal clodronate-treated group in comparison to the control group; *n* = 9, scale bar 50 μm. **c** There is a global ablation of F4/80+ cells in the liver of mice treated with liposomal clodronate, scale bar 100 μm. **d** In contrast to earlier findings, without F4/80+ cells, there is an increase in desmin+ cells post-thermal injury; *n* = 3. *P < 0.05, **P < 0.01, ***P < 0.001, ****P < 0.0001

To verify whether accumulation of myeloid lineage cells and its spatial presence in the portal field is essential for fibrosis, we treated mice with 0.1 mL/10 g of liposomal clodronate through IP injection pre and post-thermal injury. We observed less fibrosis at day 7 post-thermal injury in clodronate-treated mice in comparison to PBS treated burned mice (Fig. 3a). Ablation of macrophages was not limited to the portal field. We observed a global ablation of macrophages in the liver (Fig. 3b–d). Furthermore, when macrophages are ablated pre- and post-thermal injury, we do not observe a reduction in desmin+ cells in the liver compared to when macrophages are present (Fig. 3d). This suggests that macrophages are essential in activating hepatic stellate cells into their pro-fibrotic form.

### Liver myeloid lineage cells show a gene expression profile favoring inflammation and fibrosis

We showed that myeloid cells are critical for liver fibrosis post-thermal injury. Then, we wanted to identify genes or signaling pathways that are modulated in myeloid cells after a severe burn and identify underlying mechanisms in myeloid cells that promote fibrosis in the liver. Myeloid lineage cells tagged with EYFP were sorted via FACS from sham-treated and burned mice 7 days after injury. Gene expression analysis (Affymetrix mouse gene 2.0 st array) was performed.

**Fig. 4:**
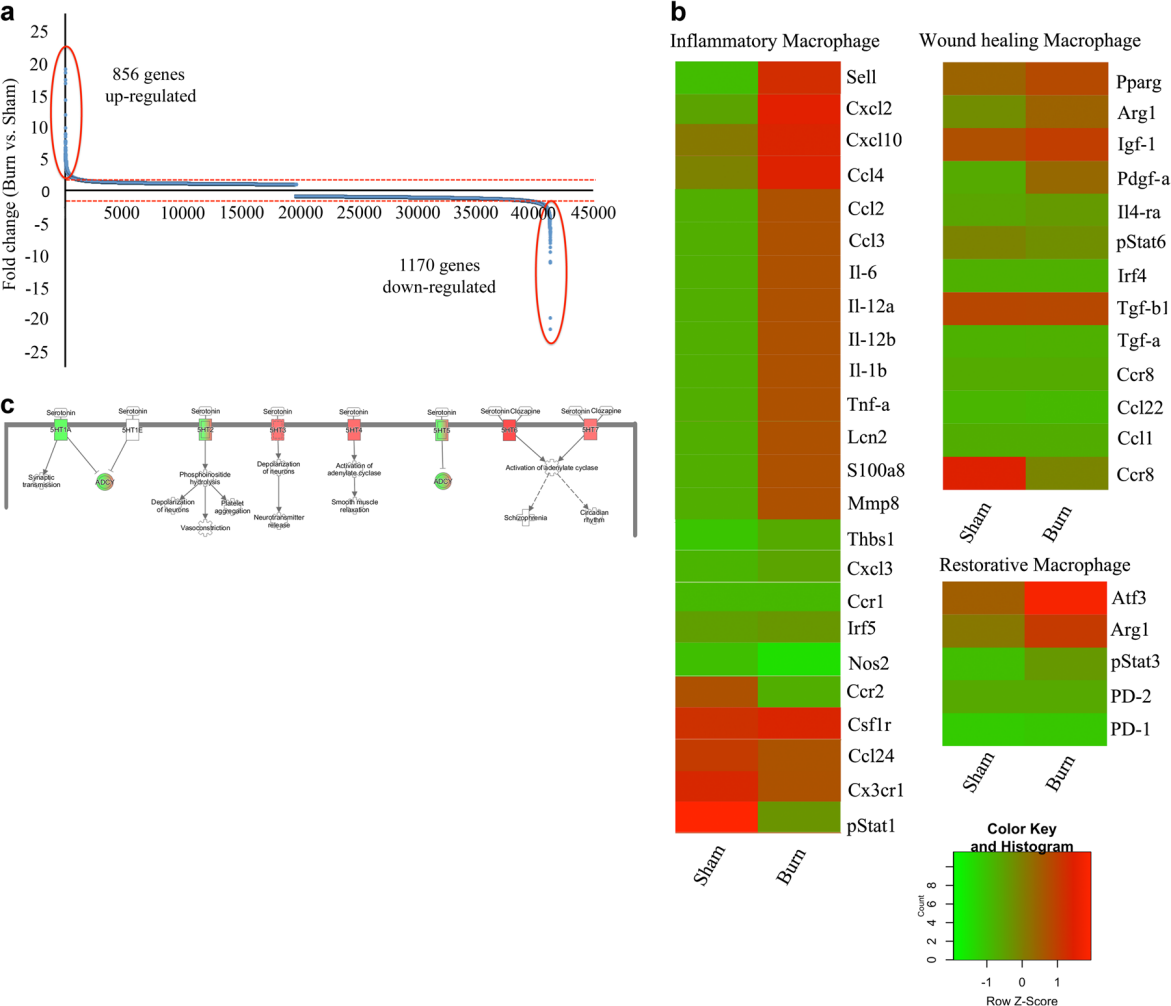
**EYFP+ cells show a gene expression profile favoring a pro-inflammatory phenotype post-thermal injury**. **a** There is a differential gene expression in ~4.5% of genes when comparing burn and sham groups. **b** Pathway analysis shows upregulation of cytokines essential for stellate cell activation and (**c**) modulation of several components of the serotonin pathway, including the 5HT_2A/2C_ receptors

Considering only genes with a fold change >2 in the analysis and comparing burn with sham samples, we observed an upregulation of 856 genes and downregulation of 1170 genes (Fig. 4a). The microarray data suggest at 7 days post-thermal injury there is upregulation of numerous genes associated with wound healing and restorative macrophages, although the majority of upregulated genes highlight a pro-inflammatory phenotype in these cells (Fig. 4b). Comparison of expression arrays (Gene Expression Omnibus database, accession no.,…) using ingenuity pathway analysis showed myeloid lineage cells were involved in numerous pathways associated with inflammation, fibrogenesis and the coagulation cascade (Supporting Fig. 3A, B). More specifically, stellate cell activation (Supporting Fig. 4A), and 5HT pathways (Fig. 4c) are some of the deregulated pathways in myeloid cells of injured animals in comparison with the sham control group.

In addition, we analyzed genes associated with inflammation and wound healing to so see if myeloid cells in the liver are polarized towards a particular phenotype 7 days post-thermal injury.

### Treating mice with the 5HT_2A/2C_ antagonist Ketanserin results in less liver fibrosis

**Fig. 5:**
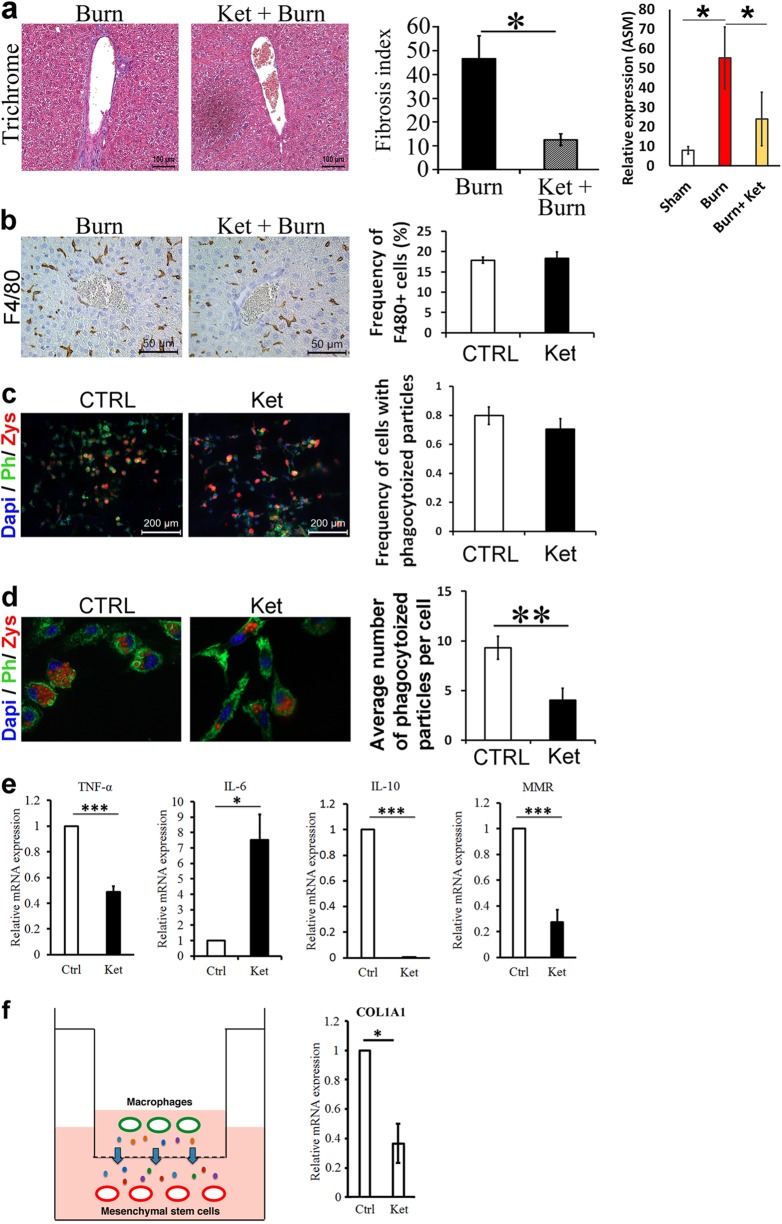
**Ketanserin decreases liver fibrosis post-thermal injury**. **a** Trichrome staining shows less fibrosis post-thermal injury in mice treated with Ketanserin and qPCR analysis shows decreased expression of ASM; *n* = 5, scale bar 100 μm; *n* = 3 per group for qPCR. **b** The accumulation and spatial pattern of macrophages do not change in the livers of mice treated with or without Ketanserin; *n* = 5, scale bar 50 μm. **c** BMDM treated with and without ketanserin and then exposed to fluorescently labeled zymosan particles show no difference in the number of macrophages, which engulf zymosan particles; scale bar 200 μm. **d** BMDM treated with Ketanserin engulfed fewer zymosan particles compared to control. **e** qPCR analysis of relative mRNA expression of M1 and M2 associated genes of BMDM from healthy mice treated with vehicle or Ketanserin. Note that expression level in treated group (Ket) divided to control for each biological replicate. **f** Co-culture of mature BMDM, pretreated with vehicle or Ketanserin, with mature BMD-MSCs shows downregulation of *Col1a1* expression in BMD-MSCs; scale bar 50 μm. *P < 0.05, **P < 0.01, ***P < 0.001

5HT has been suggested to be a key molecule in hepatic regeneration. Our pathway analysis revealed that several components of 5HT pathway have been modulated in the myeloid lineage cells post-thermal injury (Fig. 4c). Thus, we investigated its effects on liver fibrosis post-thermal injury. We observed less fibrosis in mice treated with Ketanserin (Fig. 5a) with no additional detrimental effect on the liver function according to our AST/ALT test on blood derived from thermally injured mice, mice that received Ketanserin post-thermal injury and uninjured sham control mice (Supporting Fig. 4B). To verify whether the anti-fibrotic effect of Ketanserin is due to a change in the spatial accumulation of myeloid cells in the portal field or due to change in the inflammatory profile of these cells, we quantified the number of F4/80+ cells in different zones of the liver using immunohistochemical analysis. The concentration of macrophages does not change after Ketanserin treatment (Fig. 5b). Moreover, the spatial pattern of these cells remained the same. Next, we asked if the phagocytic ability of macrophages is altered. We delivered fluorescently labeled zymosan particles to mature BMDM treated with and without Ketanserin. Although we did not observe a difference in the number of macrophages that were positive for zymosan (Fig. 5c), there was a reduction in the number of particles engulfed in macrophages treated with Ketanserin (Fig. 5d). Thus, we asked if there is an effect on the molecular signature of macrophages with Ketanserin treatment. Next, we treated BMDM with Ketanserin and looked at specific upregulated genes in the microarray in addition to key genes associated with M1 and M2 macrophages (Fig. 5e). To our surprise, in BMDM treated with Ketanserin there was a dramatic reduction in M2-associated gene IL-10 in addition to downregulation of macrophage mannose receptor (MMR) and TNF-α with up-regulation of M1 associated gene IL-6. Expression of pro-fibrotic factors TGF-β1 and TGF-β3 was not significantly different (Supporting Fig. 4C). LCN-2 showed a significant increase in BMDM treated with Ketanserin (Supporting Fig. 4C). However, we did not observe a significant difference between iNOS and Arg even though their ratio favors iNOS expression (Supporting Fig. 4C).

### Macrophages primed with ketanserin decrease pro-fibrotic characteritics of MSCs

Knowing that activated resident MSCs of the liver, i.e., stellate cells, are the main producers of collagen and macrophages being the key regulators of their activation^13^, we looked to see if BMDM primed with Ketanserin would have less of a pro-fibrotic effect on bone-marrow-derived MSCs (BMD-MSCs) of the same animal. After 3 days of co-culturing mature BMD-MSCs with mature BMDM pretreated with Ketanserin in vitro, MSCs had a decreased expression of mRNA levels of *Col1a1* compared to MSCs co-cultured with BMDM treated with vehicle as determined by qPCR analysis (Fig. 5f).

## Discussion

A severe burn injury presents with numerous challenges. Burns greater than 30% induce inflammation and hyper-metabolism, which carries on for 2 or more years after the injury, supposedly due to prolonged hepatic disturbances^25^. What may be contributing to the prolonged hepatic disturbance? As organ fibrosis is generally a chronic condition, disturbances in the functional capacity of injured organs are extended for a longer duration^26^. Liver injury, such as after a severe burn, initiates a cascade of cellular and molecular responses, which may lead to tissue fibrosis and contribute to prolonged liver dysfunction. This could be a contributing factor to the prolonged hepatic disturbance observed post-thermal injury. To the best of our knowledge, this is the first paper that addresses this pathology in the liver after a severe thermal injury. Fibrosis becomes detrimental when there is prolonged damage resulting in excessive scarring and tissue dysfunction^27,28^. Whether fibrosis in the liver after burn is beneficial or detrimental is still not known and requires further investigation.

Here, we show that there is a mild spatiotemporal pattern of liver fibrosis post-thermal injury (Fig. 1a, b). We observed fibrosis predominately around portal venules, which peaked 7–14 days’ post-thermal injury. Resolution of the excess ECM occurred by day 21 and remained low at day 42. Furthermore, there was an accumulation of EYFP+ myeloid cells in the liver post-thermal injury, which suggests there is tissue injury (Fig. 2a, b). Myeloid cells accumulated in the liver, including around portal venules, where fibrosis was observed, highlighting a role for these cells during fibrosis post-thermal injury (Fig. 2c, d). Double staining for EYFP and BrdU suggests that the increase in myeloid cells is mainly due to recruitment rather than proliferation (Supporting Fig. 1A-D), which is associated with driving the inflammatory response after tissue injury^29^. Moreover, the percentage of EYFP+ cells in the bone marrow also increase after thermal injury (Supporting Fig. 2). When we ablated macrophages using liposomal clodronate there was less hepatic fibrosis and more quiescent stellate cells relative to when macrophages are present post-thermal injury (Fig. 3). This highlights the essential role of myeloid lineage cells during liver fibrosis. While recruitment and accumulation of myeloid cells are contributing in this fibrotic response, a phenotypic change in myeloid cells can also contribute to the pathology. Profiling of myeloid cells through microarray analysis suggests that these cells exist in a spectrum of states but more directed towards a pro-inflammatory phenotype in vivo (Fig. 4b). Whereas several signaling pathways have been altered in myeloid cells in the liver post-thermal injury, we observed a significant deregulation of several components of serotonin pathway, which suggested to us that targeting this pathway might change the phenotype of myeloid cells. Treating mice with Ketanserin led to significantly less fibrosis and down regulation of hepatic alpha-smooth muscle actin (ASM) expression (Fig. 5a) and no detrimental effect on liver function (Supporting Fig. 4B), suggesting Ketanserin can be a beneficiary prophylactic. The spatial pattern or migratory capacity of macrophages, unlike the observed fibrosis, does not seem to be impaired as the concentration of macrophages does not change after Ketanserin treatment (Fig. 5b). As such, the reduction of fibrosis may be due to the impaired phagocytic ability of macrophages treated with Ketanserin (Fig. 5c, d) as phagocytosis is essential for the conversion of inflammatory macrophages to their wound healing phenotype and subsequent upregulation of their pro-fibrotic secretome^30^. This notion was supported by our in vitro experiment. At the mRNA level, we observed downregulation of TNF-α, IL-10, MMR and upregulation of IL-6 (Fig. 5e). Thus, Ketanserin appears to polarize BMDM more to a pro-inflammatory state despite their reduced phagocytic capacity. The final outcome of these changes is in the secretome, which induces less fibrosis in responsive cells (i.e., mesenchymal cells). This notion was further supported when we observed downregulation of the gene *Col1a1* in BMD-MSCs co-cultured with mature BMDMs pretreated with Ketanserin in comparison to their control group in vitro (Fig. 5f). The cytokine profile of treated BMDM appears to have changed such that BMD-MSCs have a decreased capacity in expressing *Col1a1*, which is essential for collagen production. We decided to use BMD-MSCs because we can isolate them at a much higher concentration relative to HSCs, and recent evidence shows a high level of similarity between MSCs and HSCs^31^. Stellate cells can originate from the bone marrow, similar to MSCs. Furthermore, like MSCs in other organs, stellate cells are localized around endothelial cells. HSCs are being described as the resident MSCs of the liver due to comparable gene expression profiles and inherent capacity to differentiate into adipocytes or osteocytes, and properties that promote extra-medullary hematopoiesis^32,33^. LCN-2 is a known marker for macrophage deactivation^34^ and was upregulated in macrophages conditioned with Ketanserin (Supporting Fig. 4C), which may be having a role in altering the functional capacity of macrophages in driving fibrosis. A burn injury has a profound systemic effect at different cellular and hormonal levels^35–40^. In vitro, we observe a similar response in burn-derived macrophages treated with Ketanserin relative to their sham counterparts (Supporting Fig. 4D). Thus, even in burn-derived macrophages, Ketanserin is skewing the phenotype of these cells away from their M2 “wound-healing” phenotype. However, more research is warranted on the effects of Ketanserin in vivo, As our study does not demonstrate a direct effect of macrophages on desmin-expressing cells in vivo.

Studies suggest that 5-HT has good and bad implications for the liver^41^. Treating rodents with 5HT after a 2/3 partial hepatectomy accelerated liver regeneration^18^ and boosted tissue repair after ischemia/reperfusion injury^42^. In humans undergoing liver resection, intra-platelet 5HT is shown to be associated with enhanced liver regeneration and maybe a good clinical indicator of post-operative liver dysfunction^43^. Furthermore, 5HT protects against small-for-size liver graft failure by maintaining hepatic circulation allowing for enhanced liver regeneration^44^. In aged mice, a 5HT receptor agonist rescues deficient liver regeneration^45^. Despite these promising findings, 5HT has also been shown to be a critical pro-fibrotic factor through the promotion of HSC proliferation^18^. Ketanserin, a 5HT_2A/2C_ receptor antagonist has been shown to impair HSC activation and reduce liver fibrosis^46^. However, its effect in vivo on macrophages, the regulators of HSC activation and fibrosis, is not known.

Beside a deregulation in components of serotonin pathway, our microarray analysis on isolated EYFP+ myeloid cells identified several altered signaling pathways in the livers of injured animals (Supporting Fig. 5). Farnesoid x receptor (FXR) and retinoid x receptor (RXR) pathways were three of the top five deregulated canonical pathways in myeloid cells isolated from the liver of injured animals. The farnesoid X receptor (FXR) is highly expressed in the liver and intestine. FXR has an essential role in gut-liver axis feedbacks regulating lipid and glucose homeostasis^47^. In addition, it is shown to be a powerful regulator of several hepatic metabolic pathways relevant to fatty liver disease and cholestasis^48,49^. A consequence of cholestasis is the accumulation of bile acids, which promotes inflammation and a pro-fibrogenic response. Furthermore, the forth-highest activated pathway was the coagulation system, which along with the accumulation of bile acids may explain why there is fibrosis in the liver post-thermal injury. Overall, the observed pathways suggest that myeloid cells have a diverse role in liver pathology post-thermal injury.

An increase in monocyte-derived hepatic macrophages in response to tissue injury has been well documented^50–52^. Studies suggest that pro-fibrogenic macrophages are derived from this population. These macrophages produce iNOS and are LY6C^hi^, CD11b^+^, and F4/80^+^ [30]. LY6C^hi^ monocyte-derived macrophages have been shown to be the main pro-fibrotic macrophages in numerous tissues including the liver^53–55^. Our data support the notion that accumulated myeloid cells in the liver are due to recruitment of these cells, which is associated with liver fibrosis. Furthermore, inhibiting the 5HT_2C_ receptor impairs mRNA expression of CCL2^56^, a potent cytokine for monocyte recruitment^57^. Ketanserin is a known 5HT_2A/2C_ receptor antagonist, which may consequently be impairing recruitment of pro-fibrotic LY6C^hi^ monocytes to the liver, suggesting another possible mechanism for the anti-fibrotic effect of Ketanserin in the liver.

In conclusion, we describe novel phenomena after a severe thermal injury in the liver. Whether this fibrotic response is protective or pathologic is yet to be determined. Nevertheless, we show an essential role in myeloid lineage cells mapping out fibrosis in the liver. 5HT antagonist Ketanserin appears to change the molecular signature of macrophages in a way that impairs this pro-fibrotic response. While we observe a beneficial effect of Ketanserin in inhibiting liver fibrosis, it would be essential to unravel its role on other organs, particularly in the skin, which is the first damaged organ in burn patients. However, we did not observe any change in AST/ALT, animal weight, nor general wellness of the animals between control and treated groups. If detrimental effects are observed in other organs with Ketanserin treatment, then tissue-specific delivery of Ketanserin would a safer treatment approach.

## Electronic supplementary material

Supplemental Material File #1
