# Supplementary material for: Accumulation of myeloid lineage cells is mapping out liver fibrosis post injury: a targetable lesion using Ketanserin
Source: Exp Mol Med. 2018 Jul 19;50(7):1–13. doi: 10.1038/s12276-018-0118-x (PMC6053408; doi:10.1038/s12276-018-0118-x)
Supplement: Supplementary file 1 — Supplemental Material File #1 [file 12276_2018_118_MOESM1_ESM.pdf]

Supporting Information Figure 1: Majority of EYFP +ve cells in the liver are F4/80+ve and CD11b+ve.
(**A**) FCM analysis of cells isolated from the liver is showing that the majority of EYFP+ cells in the liver are CD11b+. (**B**) Quantification of the number of F4/80+/ EYFP+ cells in the liver, showing the majority of EYFP+ cells in the liver (**C**) are F4/80+ scale bar 50μm. (**D**) Double-staining for EYFP and BrdU is showing no EYFP+ cell which is positive for BrdU in Sham and burn group (day 7) and a very limited number of EYFP+ which is positive for BrdU at day 14 post-injury. The arrow shows proliferating EYFP +ve cell; arrowhead shows proliferating EYFP –ve cell; scale bar 100μm. Note that the majority of EYFP+ cells are BrdU negative. Please note that the small red particles which are not pointed out are autofluorescence most probably due to the presence of red blood cells.

Supporting Information Figure 2: Thermal injury increases the percentage of bone marrow myeloid cells.
(**A and B**) Flow cytometry on bone marrow-derived cells shows a 50% increase in the percentage of EYFP +ve cells post-thermal injury.

Supporting Information Figure 3: Thermal injury up-regulates several pathways that associated with liver injury in the myeloid cells.
(**A**) There is an up-regulation of genes in EYFP +ve cells associated with liver dysfunction (**B**) Pathway analysis shows high up-regulation of the coagulation system in the myeloid cells.

Supporting Information Figure 4: EYFP+ve cells are skewed more towards a pro-inflammatory phenotype 1-week post thermal injury *in vivo*. (**A**) Heat map of up-regulated genes associated with inflammation and wound healing in EYFP+ cells from the livers of sham and thermally injured mice. (**B**) Measurement of liver function enzymes ALT and AST show Ketanserin is not having a detrimental effect on the liver's functional capacity. (**C**) Ketanserin does not modulate all the genes, which are associated with pro-inflammatory macrophages. (**D)** qPCR analysis of relative mRNA expression of M1 and M2 associated genes of BMDM from thermally injured mice treated with vehicle or Ketanserin. (*$P < 0.05$).

Supporting Information Figure 5: Pathway analysis shows deregulation of numerous pathways associated with inflammation and fibrosis.

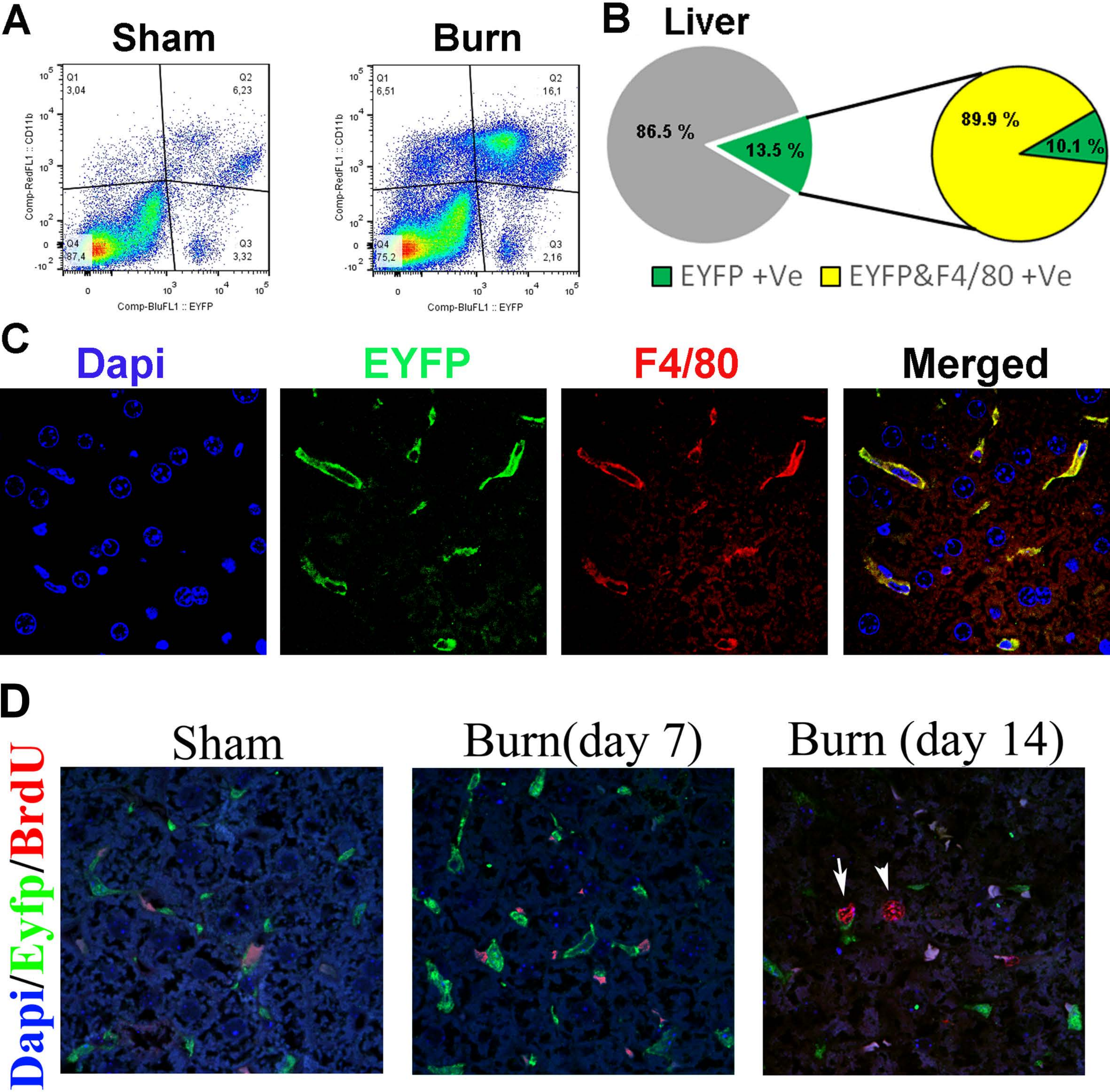
A
Sham
Burn
Q1
3,04
Q2
6,23
Q3
3,32
Q4
87,4
Q1
6,51
Q2
16,1
Q3
2,16
Q4
75,2
Comp-RedFL1 :: CD11b
Comp-BluFL1 :: EYFP
B
Liver
86.5 %
13.5 %
89.9 %
10.1 %
EYFP +Ve
EYFP&F4/80 +Ve
C
Dapi
EYFP
F4/80
Merged
D
Sham
Burn(day 7)
Burn (day 14)
Dapi/Eyfp/BrdU

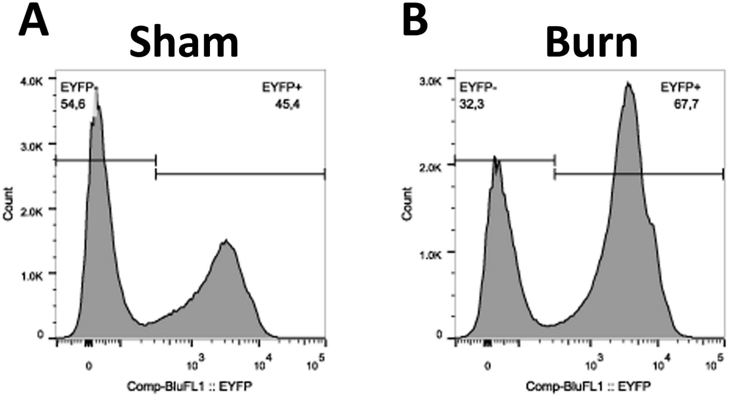

A
Sham
EYFP-
54,6
EYFP+
45,4
4.0K
3.0K
2.0K
1.0K
0
Count
0
10^3
10^4
10^5
Comp-BluFL1 :: EYFP
B
Burn
EYFP-
32,3
EYFP+
67,7
3.0K
2.0K
1.0K
0
Count
0
10^3
10^4
10^5
Comp-BluFL1 :: EYFP

A

Hepatotoxicity

Name	p-value range	# Molecules
Liver Necrosis/Cell Death	3.07E-01 - 1.82E-09	55
Liver Steatosis	3.07E-01 - 4.07E-09	58
Liver Inflammation/Hepatitis	3.84E-01 - 9.39E-09	57
Liver Damage	4.74E-01 - 3.17E-08	56
Liver Cholestasis	3.07E-01 - 6.41E-08	28

B

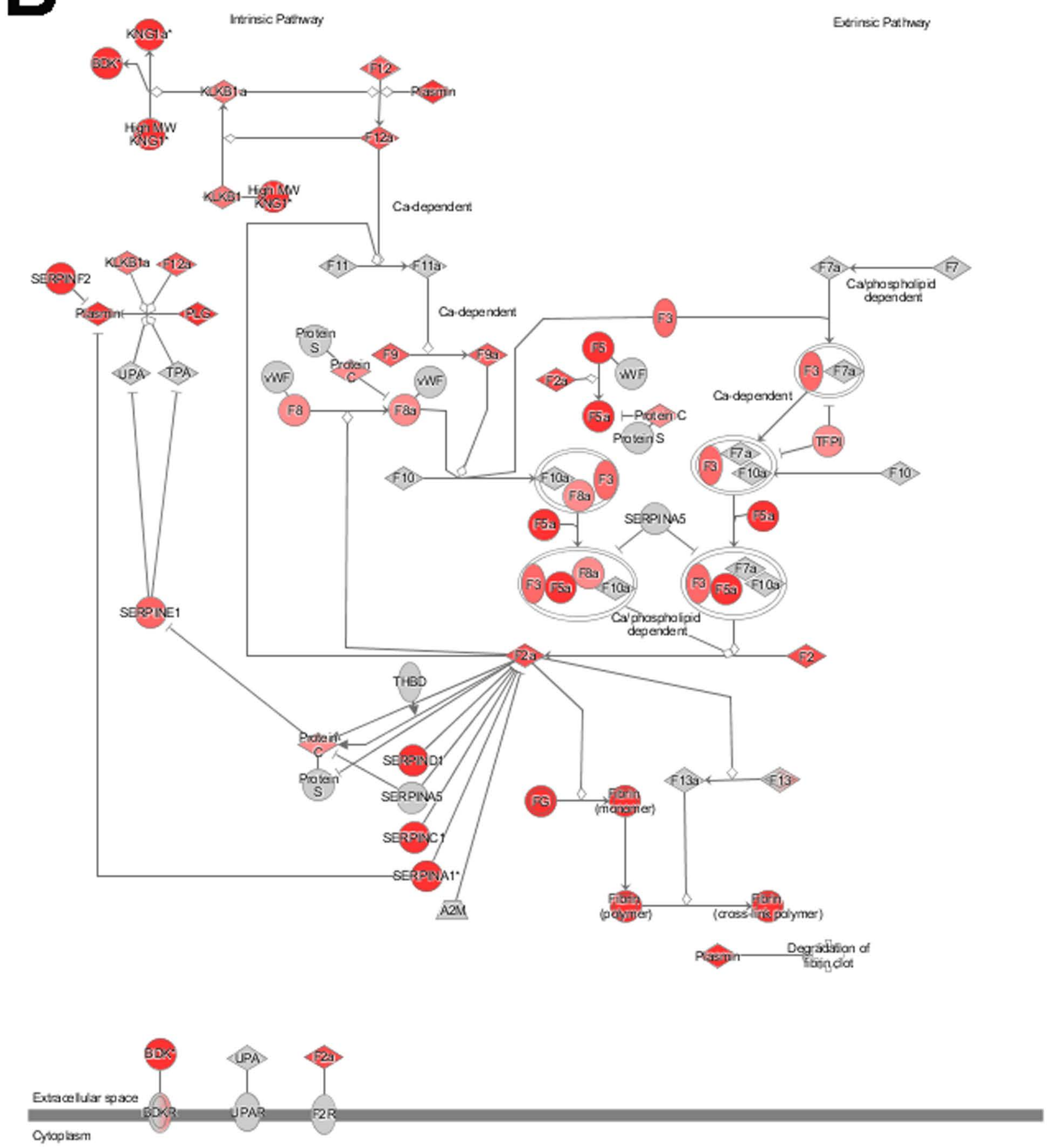

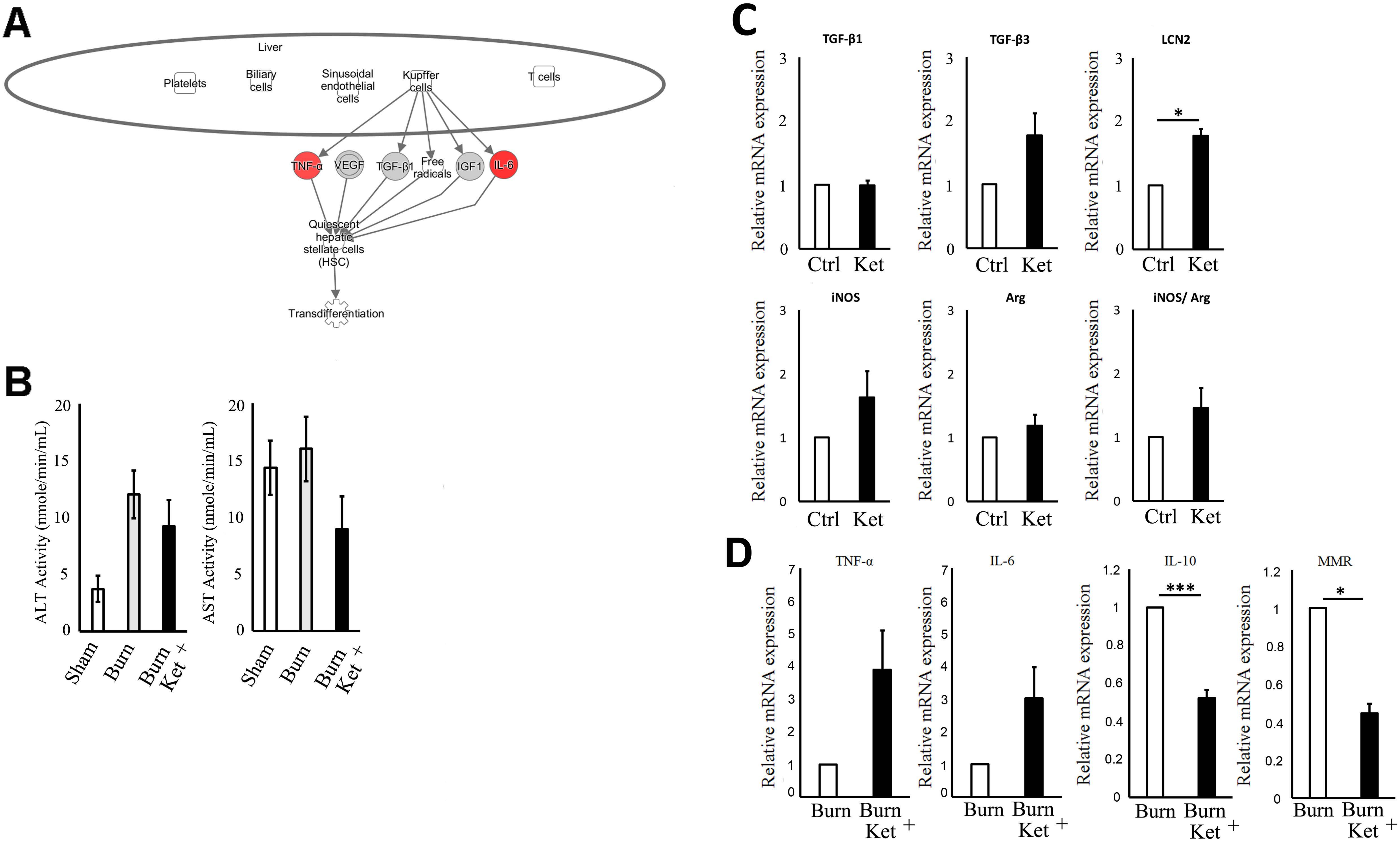
A
Liver
Platelets
Biliary cells
Sinusoidal endothelial cells
Kupffer cells
T cells
TNF-α
VEGF
TGF-β1
Free radicals
IGF1
IL-6
Quiescent hepatic stellate cells (HSC)
Transdifferentiation
B
ALT Activity (nmole/min/mL)
AST Activity (nmole/min/mL)
Sham
Burn
Burn + Ket
C
TGF-β1
TGF-β3
LCN2
iNOS
Arg
iNOS/ Arg
Relative mRNA expression
Ctrl
Ket
D
TNF-α
IL-6
IL-10
MMR
Relative mRNA expression
Burn
Burn + Ket

Analysis: Ali-1 - 2015-07-13 06:09 PM

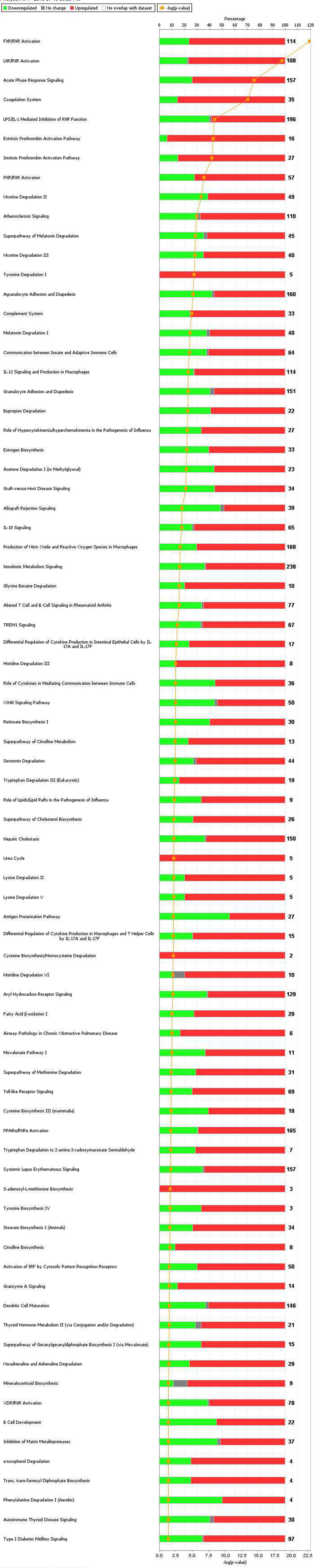

© 2000-2015 QIAGEN. All rights reserved.
